# Global Perspectives in AKI: Sri Lanka

**DOI:** 10.34067/KID.0000000000000357

**Published:** 2024-01-15

**Authors:** Eranga Sanjeewa Wijewickrama, Nalaka Herath

**Affiliations:** 1 Department of Clinical Medicine, Faculty of Medicine, University of Colombo, Colombo, Sri Lanka; 2 Nephrology Unit, Teaching Hospital, Kurunegala, Sri Lanka

**Keywords:** AKI, dialysis, epidemiology and outcomes, RRT, risk factors

## Introduction

Major advances have been made in the prevention, diagnosis, and management of AKI leading to substantial improvements in the outcomes of AKI globally. The available data on AKI primarily focuses on high-income countries while it remains scarce in low- and low–middle-income countries because of underdetection and the absence of adequate recording systems.

In this review, we aim to describe the incidence, main causes, risk factors, management, and outcomes of AKI in Sri Lanka, which is a lower middle-income country.

Sri Lanka has achieved strong health outcomes over and above what is commensurate with its income level. Most services in the country, including inpatient care (95%) and outpatient care (50%), are provided by the public sector.^1^ Curative care is provided across different levels. Preventive health care is provided through geographically defined areas, each served by a medical officer of health, who is given strong supportive supervision. The government of Sri Lanka remains a key provider of inpatient care. Medication and investigations are provided free of charge. The role of the private sector in health care is growing, but accessible only to a fraction of the population who can afford the high costs.

## Epidemiology of AKI

While the incidence and causes of hospital-acquired AKI in Sri Lanka are similar to those in other parts of the world, there is a key difference when it comes to community-acquired AKI. Unlike in high-income countries, the incidence and causes of community-acquired AKI in Sri Lanka are quite distinct. This is likely because of a variety of factors, including differences in socioeconomic status, environmental exposures, and health care access. Understanding these differences is critical for developing effective prevention and treatment strategies for AKI in Sri Lanka and other similar settings.

Sri Lanka lacks a national AKI registry, and most of the data on AKI are reported through case reports, cross-sectional studies, and small cohort studies. A study conducted in an intensive care unit (ICU) in a tertiary care hospital revealed an AKI incidence of 60.2%.^2^ The highest incidence of AKI was observed in patients admitted because of cardiac causes, followed by sepsis, respiratory, and hepatic causes. Thirty-seven percent (37%) of them were in AKI Network stage 3 and required RRT.

Another study conducted among 333 patients managed in non-ICU settings in a tertiary care center revealed that the commonest cause of AKI was systemic infections (59.5%) while nephrotoxic medications (33.6%), dehydration (24.6%), and pyelonephritis (10.8%) were the other common causes of AKI.^3^ Thirty-five percent had diabetes mellitus, 34% had hypertension, and 23.4% of patients required hemodialysis.

## Causes of Community-Acquired AKI in Sri Lanka

The predominant causes of community-acquired AKI in Sri Lanka include tropical infections; diarrheal illnesses; exposure to animal, plant, and environmental toxins; and obstetric causes (Table 1).

**Table 1:** Common causes of community-acquired AKI in Sri Lanka

Causes
**Infections**
Leptospirosis
Pyelonephritis
Dengue fever
Scrub typhus
Gastrointestinal illness/gastroenteritis
**Medications associated with AKI**
Nonsteroidal anti-inflammatory drugs
Aminoglycosides
Vancomycin
**Toxins**
Animal toxins—snakebites (*Daboia russelii*, *Hypnale* spp.)
Plant toxins—star fruit, herbal medicine
Environmental toxins—paraquat, ethylene glycol, detergents
**Obstetric causes**
Preeclampsia
HELLP syndrome
Acute fatty liver of pregnancy
Septic abortions

HELLP, Hemolysis, Elevated Liver enzymes, Low Platelets.

## Leptospirosis

Sri Lanka is categorized as leptospirosis hyperendemic with an estimated morbidity of 300.6 and mortality of 17.98 per 100,000 population per year.^4^ Development of AKI in leptospirosis, commonly associated with liver injury and myocarditis, is an indicator of severe disease leading to prolonged hospital stay, ICU care, and sometimes death. In a study conducted among hospitalized patients with serologically confirmed leptospirosis, the incidence of AKI was found to be 87%, which was associated with increased mortality.^5^ In a study conducted in Thailand, the incidence of AKI among hospitalized patients with leptospirosis was much lower at 37%, implying potential variations in host and pathogen factors involved in the development of AKI across different regions.^6^

## Snakebites

Sri Lanka is home to many species of snakes, of which four are considered highly venomous. Among these, Russell's viper (*Daboia russelii*) and hump-nosed pit viper (*Hypnale* spp.) account for almost all the cases of AKI following snakebites in Sri Lanka.^7^ AKI incidence ranges from 10% to 40% following Russell's viper bites^8^ while it is around 1%–10% following hump-nosed pit viper bites.^9^ AKI is often mild and often recovers with supportive care. However, severe AKI has been reported in 1%–3% of patients with *Hypnale* spp. envenoming and often occurs in association with hematologic markers of thrombotic microangiopathy.^9^

## Plant Toxins

Star fruit (*Averrhoea carambola*) is a popular fruit in Sri Lanka, which is valued for its medicinal and nutritive properties. The consumption of the fruit and its juice, which contains oxalic acid, leads to the development of oxalate nephropathy and AKI.^10^ This usually resolves spontaneously, but in some results in CKD. It is believed that AKI due to star fruit ingestion could be more frequent than indicated in the literature. Clinical workup for unexplained AKI in Sri Lanka should include evaluation of recent ingestion of star fruit.

## Environmental Toxins

Intentional or inadvertent exposure to toxins is a prominent public health problem in Sri Lanka, with commonly used agents including plants, such as yellow oleander, along with substances such as paracetamol, paraquat, and organophosphates. AKI is a common complication and a contributor to the increased morbidity and mortality from self-poisoning.^11^

A laundry detergent (locally known as Prinso), which contains oxalic acid and potassium permanganate, is a unique agent used for self-poisoning in the Southern parts of Sri Lanka, with its wider availability.^12^ This detergent poisoning is associated with high early mortality, and AKI is a major manifestation in individuals who survive the acute phase. The amount of ingested oxalic acid was significantly associated with adverse renal outcomes.

## Diagnosis of AKI

The diagnosis of AKI in the clinical setting is based on the changes in serum creatinine and urine output as per Kidney Disease Improving Global Outcomes criteria.^13^ Novel AKI biomarkers, such as kidney injury molecule-1, neutrophil gelatinase-associated lipocalin, and cystatin C, have not been adopted in wider clinical practice. Despite the availability of facilities to measure serum creatinine in most hospitals, the diagnosis is often delayed or missed. In a study conducted over a 3-month period in a medical ward at the National Hospital of Sri Lanka among 226 patients with AKI, the diagnosis was missed in 29% and was delayed by 48 hours in another 17%.^14^ These delays could potentially be attributed to the lack of knowledge and awareness of AKI among nurses and junior doctors, as well as the late presentation of patients.

## Prevention and Treatment of AKI in High-Risk Groups

In Sri Lanka, the medical teams working in the preventive sector conduct regular health education programs for farmers in preventing communicable diseases, such as leptospirosis. This disease is hyperendemic in the country, and as a result, oral doxycycline is provided free of charge to paddy farmers during cultivation as a prophylaxis.

In hospitals, awareness programs are conducted for health care staff for identification of high-risk patients, optimization of hydration and BP, and avoidance of nephrotoxic medications. Diabetes and other comorbidities, old age, undergoing major surgeries, and contrast administration are the main risk factors considered. Intravenous 0.9% NaCl is routinely administered before and after contrast procedures for individuals with CKD. Oral N-acetyl cysteine is used in some centers for 48 hours despite the lack of evidence on its benefit. Optimization of hydration and BP and withdrawal of nephrotoxic medications and timely RRT are considered mainstay of treatment of AKI. Early stages of AKI in hospitalized patients are predominantly managed by physicians who are specialized in internal medicine, and nephrologists are generally consulted when the AKI fails to recover or need specialized treatment.

## RRT in AKI

**Figure 1:**
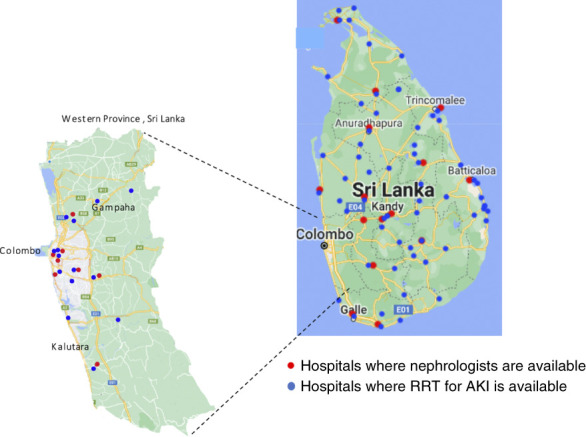
Distribution of hospitals in Sri Lanka where RRT and nephrologists are available.

RRT for AKI in Sri Lanka is available at kidney units, satellite dialysis units, and ICUs in Sri Lanka. Kidney units are primarily located in larger tertiary referral centers (national hospitals, teaching hospitals, provincial and district general hospitals) (Figure 1). Satellite dialysis units are located at some of the base hospitals. Acute hemodialysis, continuous RRT (CRRT), and slow low-efficiency dialysis are available in all kidney units. Satellite dialysis units primarily provide acute hemodialysis. Most ICUs are equipped with the necessary facilities to administer CRRT and slow low-efficiency dialysis. Acute peritoneal dialysis (PD), using flexible catheters, is performed in some pediatric ICUs. The use of rigid PD catheters for acute PD is now rare in Sri Lanka because of the high incidence of complications associated with it.

Most of the patients with AKI admitted to hospitals are initially managed by physicians specialized in internal medicine. After initial assessment and treatment, they are referred to nephrologists. In places where nephrologists are not available, physicians obtain advice through telephone. Patients needing specialized treatment are transferred to tertiary care centers for further care. Prescription and supervision of intermittent hemodialysis are performed by nephrologists except in institutions where nephrologists are not available, where the treatment is prescribed by physicians specialized in internal medicine. Continuous modalities are prescribed and managed jointly by both critical care specialists and nephrologists. Patients with AKI in ICUs are managed with either intermittent or continuous modalities on the basis of the availability of the treatment, hemodynamic stability of the patient, and the presence of other comorbidities and complications. RRT (both continuous and intermittent) is funded by the Ministry of Health and is accessible to all patients free of charge.

## Outcomes of AKI

The data on outcomes of AKI in Sri Lanka is lacking. Few small observational studies have reported short-term outcomes of AKI. In one such study conducted in a tertiary care center that included 333 hospitalized non-ICU patients with AKI, complete kidney recovery was observed in 46.8% at discharge,^3^ 20% needed RRT, and 12% died before discharge. In nearly 50% of this patient population, sepsis was determined as the cause for the AKI, and 70% had severe AKI at the time of referral to the nephrology services, which explains the high mortality in this group. Eleven percent had CKD during follow-up on 90 days. In another study that included 68 patients with AKI, 29.4% made complete kidney recovery while 41.2% made partial recovery at discharge and the mortality rate was 24%.^15^ 85.3% of these patients required RRT, of which 79.3% received hemodialysis while 20.7% received acute PD.

## Follow-Up Care in AKI

In the hospital setting, patients receiving care from nephrologists typically continue to be followed up by them post-hospitalization. However, patients who are solely managed by physicians specialized in internal medicine during their hospital stay are referred to outpatient kidney clinics if there is incomplete kidney recovery.

## Challenges and Solutions in the Management of AKI in Sri Lanka

Sri Lanka has 1.6 nephrologists per million population,^16^ which is highly inadequate to maintain the standard kidney care. This compares with 7.4 and 20.1 nephrologists per million population for the United Kingdom and Europe, respectively.^17^ Migration of professionals because of the current economic crisis has exacerbated the existing shortage in human resources. Furthermore, there is a poor uptake of the specialty because of longer duration of training. Exploring strategies to reduce the training period could potentially enhance the appeal of the specialty among trainees, addressing the scarcity of professionals in the field.

The prevailing economic crisis has led to recurrent disruptions in the supply of consumables required for CRRT, thereby hindering the continuous provision of this treatment to hemodynamically unstable patients. To address these shortages in the long run, it is suggested to establish and support local manufacturing facilities for CRRT solutions. This approach would not only provide the necessary resources but also promote self-sustainability within the region.

The lack of knowledge of AKI among the health care staff is a major challenge that leads to delays in the identification of high-risk patients for AKI and the detection of AKI. Then, patients with AKI are often referred late to the nephrology services. Furthermore, some patients seek treatment for AKI from traditional healers who reside in the local community, leading to significant delays in presenting to standard health care institutions. Expansion of public awareness programs with a focus on prevention of common causes of community-acquired AKI including nephrotoxic drugs, volume depletion, endemic infections, and toxic exposures has the potential to significantly reduce the incidence of preventable AKI. There is a need to strengthen the education of AKI evaluation and management within undergraduate medical curricula and during internal medicine training, as well as through continuing medical education programs designed for practicing physicians and nurses conducted by the Ministry of Health, medical universities, and national medical societies.

Drug level monitoring for potentially nephrotoxic antibiotics, such as vancomycin, gentamicin, and amikacin, is not routinely available. As a result, many patients are at risk of developing potentially preventable AKI after administration of these antibiotics. Given the current economic crisis in Sri Lanka, which makes the development of such facilities unlikely in the near future, it is imperative to focus on developing guidelines for the local setting regarding the utilization and dosage adjustment of such medications.

Self-poisoning, which is a common cause of AKI, has been a significant public health issue in Sri Lanka. The government, along with various organizations, has implemented several initiatives to reduce self-poisoning rates and improve mental health support. Some of these initiatives include banning of several highly toxic weedicides such as paraquat, imposing laws and regulations to restrict access to pesticides, establishing poison control centers to provide emergency medical advice and support for cases of poisoning, expanding mental health facilities, and providing training to health care professionals to better identify and manage mental health issues and instigating various community-based programs to raise awareness about mental health and self-harm prevention.

There is a clear need for further improvement in the reporting and recording of AKI in Sri Lanka. Establishment of an AKI registry would be helpful for resource allocation and tracking practice patterns in the management of AKI in Sri Lanka.

AKI leads to considerable morbidity and mortality in Sri Lanka. Identification of individuals who are at high risk of developing AKI is important for its prevention, early diagnosis, and proper treatment. The limitations in the infrastructure, manpower, local research, and reporting and recording of AKI are key challenges in providing optimal care for AKI in Sri Lanka.
